# Identification and Evaluation of Plasma MicroRNAs for Early Detection of Colorectal Cancer

**DOI:** 10.1371/journal.pone.0062880

**Published:** 2013-05-14

**Authors:** Xiaoya Luo, Christian Stock, Barbara Burwinkel, Hermann Brenner

**Affiliations:** 1 Division of Clinical Epidemiology and Aging Research, German Cancer Research Center (DKFZ), Heidelberg, Germany; 2 Department of Gastroenterology, Beijing Friendship Hospital Affiliated with the Capital Medical University, Beijing, China; 3 Divison of Molecular Biology of Breast Cancer, Department of Gynecology and Obstetrics, University Heidelberg, Heidelberg, Germany; 4 Molecular Epidemiology Group, German Cancer Research Center (DKFZ), Heidelberg, Germany; University of Bari & Consorzio Mario Negri Sud, Italy

## Abstract

**Background:**

Colorectal cancer (CRC) is one of the most commonly diagnosed cancers. Circulating microRNAs (miRNAs) have been suggested as potentially promising markers for early detection of CRC. We aimed to identify and evaluate a panel of miRNAs that might be suitable for CRC early detection.

**Methods:**

MiRNAs were profiled by TaqMan MicroRNA Array and screened for differential expression in 5 pools of plasma samples of CRC patients (N = 50) and 5 pools of neoplasm-free controls (N = 50). Additional miRNAs were selected from a literature review. Identified candidates were evaluated in independent validation samples with respect to discrimination of CRC patients (N = 80) or advanced adenoma patients (N = 50) and neoplasm-free controls (N = 194). Diagnostic performance of the panel of miRNAs was assessed by multiple logistic regression, using bootstrap analysis to correct for over-optimism.

**Results:**

Five miRNAs identified to be differentially expressed from TaqMan MicroRNA Array (miR-29a, -106b, -133a, -342-3p, -532-3p), and seven miRNAs reported to be differentially expressed in the literature (miR-18a, -20a, -21, -92a, -143, -145, -181b) were selected for validation. Nine of the twelve miRNAs (miR-18a, -20a, -21, -29a, -92a, -106b, -133a, -143, -145) were found to be differentially expressed in CRC patients and controls in the validation samples. The optimism-corrected area under the curve was 0.745 (95% confidence interval: 0.708–0.846). None of the selected miRNAs showed significant differential expression between advanced adenoma patients and neoplasm-free controls.

**Conclusion:**

The identified panel of miRNAs could be of potential use in the development of a multi-marker blood based test for early detection of CRC. Impact: The study underscores the high potential of plasma miRNAs for the improvement of current offers of non-invasive CRC screening.

## Introduction

With over 1.2 million new cases and 608,700 deaths per year, colorectal cancer (CRC) is the third most commonly diagnosed cancer in males and the second in females, and the fourth most common cause of death from cancer worldwide [1]. Due to its typically very slow development over many years, perspectives for early detection are much better than for many other forms of cancer. It has been estimated that over 95% of cases of CRC would benefit from curative surgery if diagnosis was made at an early or premalignant polyp stage [2], [3]. A number of early detection procedures have been developed and are increasingly applied, including endoscopic examinations, stool- and blood-based tests. Blood based tests would appear to be particularly attractive as they are minimally invasive and might receive high levels of adherence when applied as primary screening tests in population based screening. A large number of blood markers have been proposed and evaluated, including protein, cytological, mRNA, and DNA markers [4], [5], but diagnostic performance has mostly been insufficient for application as a primary tool in population-based screening. Furthermore, most studies relied on small convenience samples from clinical settings, and rather promising results from small studies have often not been replicated in subsequent larger scale validations.

MicroRNAs (miRNA) are 18∼22 nucleotide non-coding RNAs that post-transcriptionally regulate gene expression and control various cellular mechanisms [6]. There is increasing evidence that miRNAs are widely dysregulated in cancer and may have potential application for cancer diagnosis, prognosis and treatment [7]. Furthermore, recent development of miRNA microarrays has made large profiling studies in cancer patients possible.

The stability of cell-free miRNAs in body fluids enables circulating miRNAs to be potential biomarkers for noninvasive diagnosis of cancer and other disease [8]. Several recent studies found some circulating miRNAs, such as miR-29a, miR-92a and miR-221, to be differentially expressed in CRC patients and therefore to be of potential use as non-invasive biomarkers for CRC screening [9]–[11].

The aim of this study was to identify and evaluate a panel of plasma miRNAs that might serve as biomarkers for the early detection of CRC.

## Materials and Methods

### Study Design and Study Population

Cases with sporadic CRC were recruited prior to initiation of therapy at the University Clinic of Heidelberg in the context of the DACHS+ study, which is a satellite study to DACHS, an ongoing case-control study conducted in the Rhine-Neckar region of Germany [12], [13].

Patients with advanced colorectal adenomas and controls free of colorectal neoplasms were randomly selected from participants of screening colonoscopy recruited in the BLITZ study, an ongoing study designed to evaluate novel promising markers for early detection of CRC and previously described in detail elsewhere [14]–[16]. Briefly, patients were recruited, and blood samples were taken at gastroenterologists’ offices at a preparatory visit, typically about one week prior to screening colonoscopy.

Both the DACHS+ and the BLITZ study were approved by the ethics committees of the Medical Faculty at the University of Heidelberg and of the Medical Boards of Baden-Wuerttemberg and Rhineland-Palatinate. Written informed consent was obtained from each participant.

Our investigation involved two main phases, a marker identification phase and a marker validation phase:

In the marker identification phase, promising miRNAs were screened by TaqMan MicroRNA Array in pooled plasma samples from 50 CRC patients and 50 neoplasm-free controls, using five pools of plasma samples from ten CRC patients each, and five pools of plasma samples from ten neoplasm-free controls each. In addition, seven miRNAs described to be differentially expressed in CRC cases and controls in previous publications were identified from a systematic literature review (miR-18a, -20a, -21, -92a, -143, -145, -181b) [17].

In the marker validation phase, expression of the identified miRNAs was evaluated in independent samples of (i) 80 CRC cases and 144 controls free of colorectal neoplasms and (ii) 50 patients with advanced adenomas and 50 controls free of colorectal neoplasms.

### Laboratory Procedures

#### (i) Sample preparation and RNA extraction

The blood samples were collected in EDTA tubes. Blood samples from cancer patients were taken before surgery or any other therapy and blood samples from participants who underwent screening colonoscopy were taken prior to colonoscopy. The blood samples were centrifuged at 2123g for 10 min at 4°C and the supernatant was transferred into new tubes. Plasma samples were stored at −80°C until use. Total RNA containing small RNA was extracted from 200 µl (samples used in the identification phase) or 115 µl (samples used in the validation phase) plasma using a combination of Trizol LS reagent (Invitrogen, Carlsbad, California, USA) and miRNeasy Mini Kit (Qiagen, Hilden, Germany) as well as 5 fmol/µl cel-miR-39 as external preparation control as described before [18]. Samples were eluted in a final volume of 30 µl.

#### (ii) MicroRNA profiling from plasma samples

Profiling was performed using TaqMan MicroRNA Array (Applied Biosystems, Foster City, California, USA) which enables quantification of 667 human microRNAs.(Table S1) Megaplex reverse transcription reaction and pre-amplification reaction were performed by G-Storm GS2 Thermal Cycler (G-Storm, UK). Real-time quantitative polymerase chain reaction (qRT-PCR) was performed using 7900HT Fast Real-Time PCR System (Applied Biosystems). The cycle threshold (Ct) is defined as the number of cycles required for the fluorescent signal to cross the threshold in qRT-PCR. Raw Ct values were calculated using the SDS software version 2.2 applying automatic baseline settings and a threshold of 0.1 was set. Only miRNAs whose Ct value was equal to or below 33 in at least either the case or the control group were taken into account for further data analysis. Data normalization was done as described by Kroh et al. [19].

#### (iii) Real time quantitative PCR verification

Selected miRNAs were measured using TaqMan MicroRNA Assays (Applied Biosystems) according to the manufacturer’s protocol. Triplicates of qRT-PCR of each sample were performed using LightCycler 480 Real-time PCR system (Roche Applied Science, Germany). ΔCt was calculated by subtracting the Ct values of internal controls from the Ct values of the miRNAs of interest, and mean ΔCt values were compared between cases and controls. Multiplex assays were performed using predefined pools of RT-primers (Table S2). The efficiency of each miRNA’s assay was determined by constructing a standard curve using a series of total RNA dilutions. All assays showed good linearity (R^2^>0.96) between the Ct values and the log of the starting quantity of total RNA of each dilution (data not shown).

### Statistical Analyses

MiRNA expression levels were compared between CRC patients and neoplasm-free controls and between advanced adenoma patients and neoplasm-free controls using the Wilcoxon-Mann-Whitney-Test (hereafter: Wilcoxon test). The comparison of miRNA expression levels between CRC patients and neoplasm-free controls in the marker identification phase was performed using the exact version of the Wilcoxon test. All tests were two-sided and P values of 0.05 or less were considered to be statistically significant.

Multiple logistic regression was employed to assess the joint use of the identified panel of miRNAs in predicting CRC in the marker validation phase. Receiver operating characteristic (ROC) curves were constructed and areas under the ROC curves (AUCs) were calculated both from unadjusted (apparent) and adjusted (“optimism-corrected”) estimates to assess discrimination of the prediction model between patients with and without CRC. The.632+ bootstrap method (with 1000 replicates) was used to adjust for overfitting of the apparent misclassification error and over-estimation of AUCs by the unadjusted estimates [20], [21]. 95% confidence intervals (CI) for the adjusted and unadjusted estimates were obtained by ordinary bootstrap analyses (with 1000 replicates).

The correlation of plasma miRNA expression levels across the 324 participants of the validation phase was assessed by Spearman correlation coefficients.

SAS version 9.2 (SAS Institute Inc., Cary, North Carolina, USA) was used to conduct Wilcoxon tests and R version 2.15.0 (R Foundation for Statistical Computing, Vienna, Austria) was used to conduct all other analyses. The R-packages “Daim” and “boot” were employed to perform bootstrap analyses [22], [23].

## Results

### Study Population

A total of 424 individuals were included (130 CRC patients, 50 advanced adenoma patients and 244 neoplasm-free controls) in the study. Demographic characteristics of the study population and distribution of tumor stages are summarized for the marker identification phase and the marker validation phase in Table 1.

**Table 1:** Study population.

Characteristic	Marker identification phase		Marker validation phase				
	(pooled samples)		(individual samples)				
	CRC patients	Neoplasm-free controls	CRC patients	Neoplasm-free controls	Advanced adenoma patients	Neoplasm-free controls	
	n = 50	n = 50	n = 80	n = 144	n = 50	n = 50	
Age (years)							
Mean±SD	67.1±11.0	61.7±6.4	68.0±10.4	62.5±7.5	65±7.6	61.6±6.3	
Gender							
Male	25	25	45	60	32	20	
Female	25	25	35	84	18	30	
AJCC stage							
I	15	–	22	–	–	–	
II	12	–	25	–	–	–	
III	20	–	26	–	–	–	
IV	3	–	5	–	–	–	
Not specified	0	–	2	–	–	–	
Tumor location							
Proximal colon	18	–	23	–	26	–	
Distal colon and rectum	32	–	56	–	22	–	
Not specified	0	–	1	–	2	–	

Abbreviations: CRC, colorectal cancer; SD, standard deviation.

### Identification of Differentially Expressed miRNAs in CRC Patients and Neoplasm-free Controls

In microarray analyses, five miRNAs were found to be statistically significantly over-expressed in the plasma of CRC patients compared to neoplasm-free controls (miR-29a: p = 0.016, miR-106b: p = 0.008, miR-133a: p = 0.032, miR-342-3p: p = 0.008, miR-532-3p: p = 0.016, exact Wilcoxon test).

### Evaluation of Internal Controls for Real Time Quantitative PCR

In our microarray data we observed no significant difference in terms of the Ct values of miR-188-5p (p = 0.83, Wilcoxon test) and miR-16 (p = 0.40, Wilcoxon test) between CRC and neoplasm-free controls. RNU6B and miR-16 have been the most widely-used endogenous control miRNA for qRT-PCR in the miRNA studies [8]. Therefore, the named three miRNAs were considered as internal controls for miRNA quantification. However, expression levels of RNU6B and miR-188-5p in the plasma were too low to be quantified by TaqMan MicroRNA Assay (mean Ct values were greater than 35). Hence, miR-16 was selected as the internal control as it showed high abundance and less variability in expression. No significant difference was observed in terms of Ct values of miR-16 (p = 0.40, Wilcoxon test) between CRC and neoplasm-free samples.

### Validation in an Independent Sample of CRC Patients and Neoplasm-free Controls

To confirm the five miRNAs (miR-29a, -106b, -133a, -342-3p, -532-3p) identified in the microarray analysis and another seven miRNAs (miR-18a, -20a, -21, -92a, -143, -145, -181b) which were previously reported to be differentially expressed in CRC (except miR-92a, they were all from studies based on tissue samples) [17], qRT-PCR was performed to analyze the expression of the selected miRNAs in our validation set (224 plasma samples in total from 80 CRC patients and 144 neoplasm-free controls).

#### (i) Expression of miRNAs

**Figure 1:**
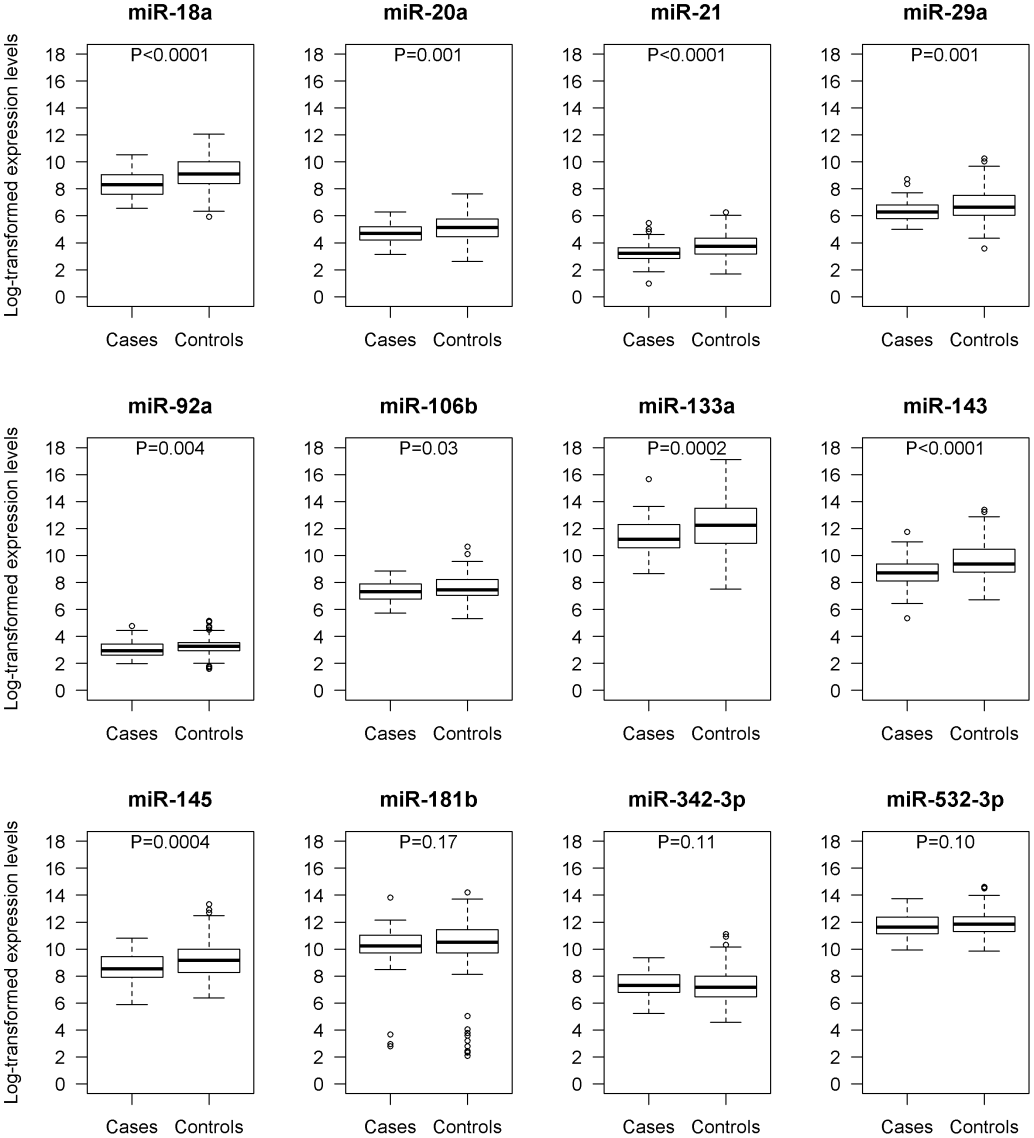
Expression levels of microRNAs in 80 colorectal cancer patients and 144 neoplasm-free controls (normalized to miR-16, log2 scale at y-axis). Box plots with smallest observation, lower quartile, median, upper quartile and largest observation are shown. The whiskers extend to the observations which are no more than 1.5 times the length of the box (interquartile range) away from the box. More extreme observations are considered outliers. P values are based on Wilcoxon tests.

Nine miRNAs (miR-18a, -20a, -21, -29a, -92a, -106b, -133a, -143, -145) showed higher expression in the plasma of CRC patients than in neoplasm-free controls (miR-18a: p<0.0001, miR-20a: p = 0.001, miR-21: p<0.0001, miR-29a: p = 0.001, miR-92a: p = 0.004, miR-106b: p = 0.028, miR-133a: p = 0.0002, miR-143<0.0001, miR-145: p = 0.0004, Wilcoxon test). No miRNA was found to be downregulated. The corresponding expression levels in plasma of CRC patients and neoplasm-free controls (normalized to miR-16) are shown in Figure 1.

#### (ii) Relationship between miRNAs and clinicopathological features of CRC patients

We examined the association between miRNA expression levels and some clinicopathological features. MiR-181b’s expression level was higher in patients with lymph node metastasis than in patients without lymph node metastasis (p = 0.024, Wilcoxon test). None of the miRNAs showed any association with tumor site or tumor stage. Likewise, no associations were seen with age, neither in cases nor in neoplasm-free controls (data not shown).

To evaluate whether the expression levels of the investigated miRNAs are associated with the development of CRC, patients were stratified by AJCC stages. In general, expression levels were very similar in early and late stage cancers. MiR-18a, 20a, 21, 29a, 133a, 143 and 145 were found to be over-expressed in plasma of CRC patients at early stages compared with neoplasm-free controls. MiR-18a, -20a, -21, -92a, -133a, -143, -145 and -181b showed higher expression levels in plasma of CRC patients at late stages compared with neoplasm-free controls. MiR-181b showed higher expression in the plasma of late stage CRC patients than early stage CRC patients (Table 2).

**Table 2:** MicroRNAs differently expressed between stage stratified colorectal cancer patients and neoplasm-free controls.

	Mean ΔCt* ofCRC patients atearly stages(stage I and II)	Mean ΔCt* ofCRC patients atlate stages(stage III and IV)	Mean ΔCt* ofneoplasm-freecontrols	P values†		
				CRC at earlystage vs controls(47 vs 144)	CRC at latestage vs controls(31 vs 144)	CRC at early stage vsCRC at late stage(47 vs 31)
miR-18a	8.36	8.48	9.17	<0.001	0.001	0.491
miR-20a	4.73	4.73	5.11	0.012	0.022	0.984
miR-21	3.24	3.27	3.80	0.001	0.002	0.878
miR-29a	6.30	6.40	6.81	0.003	0.055	0.328
miR-92a	3.08	3.01	3.29	0.058	0.013	0.597
miR-106b	7.33	7.36	7.62	0.088	0.128	0.984
miR-133a	11.44	11.46	12.31	0.003	0.012	0.935
miR-143	8.63	8.93	9.61	<0.001	0.007	0.210
miR-145	8.53	8.65	9.26	0.003	0.029	0.711
miR-181b	10.36	9.80	10.16	0.932	0.035	0.024
miR-342-3p	7.53	7.23	7.24	0.052	0.777	0.174
miR-532-3p	11.76	11.62	11.92	0.445	0.084	0.405

* Data were normalized to miR-16.

† P values are based on Wilcoxon tests.

Abbreviations: CRC, colorectal cancer.

#### (iii) Multivariate analysis

**Figure 2:**
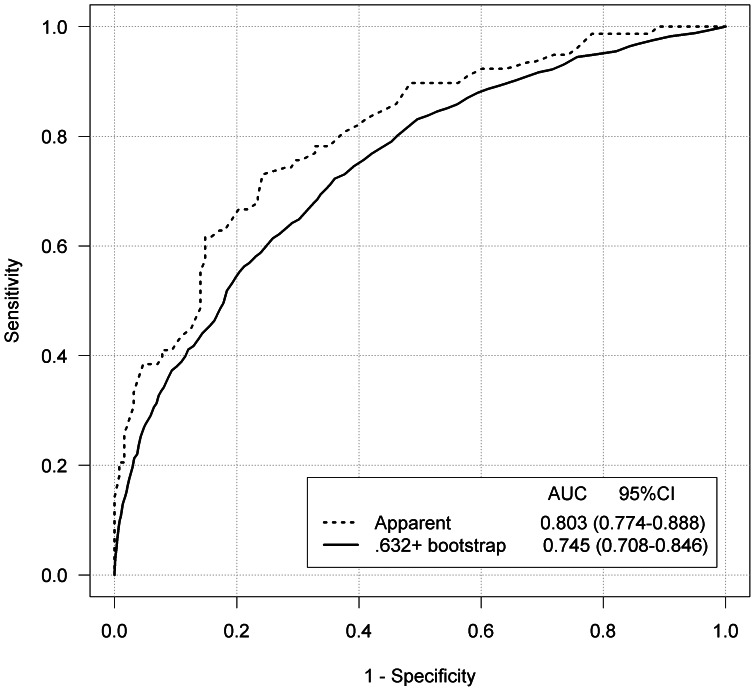
Receiver operating characteristic curves using the panel of 12 selected microRNAs for discrimination of 80 colorectal cancer patients and 144 neoplasm-free controls. Adjustment for over-optimism was done by the.632+ bootstrap method. Abbreviations: AUC, area under receiver operating characteristic curve.

With the panel of 12 miRNAs (miR-18a, -20a, -21, -29a, -92a, -106b, -133a, -143, -145, -181b, -342-3p, -532-3p), the ROC analyses yielded an unadjusted AUC of 0.803 (95% CI: 0.774–0.888) and an adjusted, i.e. optimism-corrected, AUC of 0.745 (95% CI: 0.708–0.846). The ROC curves are depicted in Figure 2. For each miRNA, the ROC curves are depicted in Figure S1.

### Expression of miRNAs in Advanced Adenoma Patients and Neoplasm-free Controls

Advanced colorectal adenomas represent a precursor stage of CRC. To assess potential use for early detection even of advanced adenomas, the identified 12 miRNAs were determined by qRT-PCR in plasma samples from 100 study participants (50 with advanced adenoma and 50 neoplasm-free controls). No statistically significant differences in miRNA expression levels were observed in the plasma of advanced adenoma patients and neoplasm-free controls.

### Correlations of Expression Levels of Plasma miRNAs

Among participants of the validation phase, strong correlations were observed between expression levels of miR-18a and miR-20a (r = 0.800, p<0.001), which are both encoded in the miR-17-92 cluster; miR-143 and miR-145 (r = 0.762, p<0.001) which are both encoded in the miR-143/145 cluster; and miR-29a and miR-106b (r = 0.694, p<0.001), two closely residing miRNAs on chromosome 7q. The expression levels of some other miRNAs which are not colocated in the genome were also highly correlated. On the other hand, miR-92a which is also encoded in miR-17-92 cluster was not highly correlated with miR-18a or miR-20a (r = 0.340 and 0.251 respectively) (Table S3).

## Discussion

In this study, we compared the expression levels of twelve miRNAs in plasma samples of CRC patients and neoplasm-free controls. To our knowledge, this is the first report on circulating miRNA for CRC detection using TaqMan MicroRNA Array for miRNA profiling. Five miRNAs (miR-29a, -106b, -133a, -342-3p, -532-3p) were identified from the microarray analyses and seven were selected from the literature (miR-18a, -20a, -21, -92a, -143, -145, -181b) for further investigation [17]. In our validation study, the expression levels of miR-18a, -20a, -21, -29a, -92a, -106b, -133a, -143, and -145 were found to be significantly higher in CRC patients than in neoplasm-free controls. ROC analysis of the identified miRNAs adjusted for over-optimism by bootstrap yielded an AUC of 0.745 (95% CI: 0.708–0.846). This result compares favorably with results of other blood-based tests for CRC detection [5].

Diagnostic characteristics of circulating miRNAs for CRC were evaluated previously in two recent studies. Ng et al. reported 0.89 sensitivity and 0.70 specificity at a certain cut-off point of miR-92a, and an AUC of 0.885 (95% CI: 0.83–0.94) based on 90 CRC patients (TNM stage I/II/III/IV: 6/34/23/27) and 50 controls [10]. Huang et al. reported 0.830 sensitivity and 0.847 specificity, and an AUC of 0.883 (95% CI: 0.830–0.937) obtained in a multivariate analysis, including miR-29a and miR-92a based on 100 CRC patients (TNM stage I/II/III/IV: 27/25/38/10) and 59 controls [9]. Thus, both studies found higher AUC estimates than obtained in the present study. These studies had not adjusted for over-optimism. While the 95% confidence interval (0.774–0.888) of the unadjusted AUC in our study encompassed the AUCs reported by Ng et al. and Huang et al., adjustment for over-optimism attenuated the AUC by approximately 6 percent units. Nevertheless, even our unadjusted AUC estimate was slightly lower than the previous reported ones, despite the larger number of included miRNAs. Factors that could partly account for these differences include stage distribution of CRCs and chance. The percentage of early stage CRC patients (AJCC stage I and II) was higher in our study (59%) than in the previously reported studies (44% and 52%, respectively) and probably closer to the stage distribution expected in a screening setting [5]. However, no major difference of expression levels by stage was observed in our study.

Colorectal adenomas represent a precursor stage of adenocarinoma. A previous study had reported that the differential expression of plasma miR-29a and miR-92a could also discriminate advanced adenoma patients from neoplasm-free controls even though discrimination was less pronounced than for CRC patients [9]. In our study, none of the investigated miRNAs was found significantly differentially expressed in the advanced adenoma patients compared to the neoplasm-free controls. These results suggest that the investigated miRNAs may not be useful for the diagnosis of advanced adenomas. However, lack of significant differences may also be due to limited power to detect moderate differences with the the sample size of our substudy comparing carriers of advanced adenomas and neoplasm-free subjects Given the observed lack of differential expression of individual miRNAs, a multivariate model for prediction of advanced adenomas was not applied.

Although the differential expression of plasma miR-18a, -20a, -21, -106b, -133a, -143, -145 and -181b in CRC patients was examined in some tissue sample based studies [24]–[27], this is, to our knowledge, the first study reporting on the expression of these miRNAs in plasma samples of a large number of patients with CRC and neoplasm-free controls. Statistically significant differences in the expression of all analyzed miRNAs except miR-181b, miR-342-3p and miR-532-3p were observed. Intriguingly, our data showed that the expression levels of miR-133a -143, and -145 were significantly over-expressed in the plasma of CRC patients compared to the neoplasm-free controls, which seems to contradict data consistently showing less expression of these three miRNAs in cancer in tissue sample based studies [28]–[34]. Further studies with larger numbers of patients and simultaneous measurements of miRNA expression in plasma and tissue may be warranted to clarify this issue.

A common problem in research concerning circulating miRNAs is that no consensus internal control has been established. We evaluated miR-16, miR-188-5p and RNU6B, and due to the consistent, stable and high expression across all plasma samples, miR-16 was selected as internal control in the plasma sample based tests, but more empirical validations are still needed for a consensus on robust and accurate internal controls.

The nine miRNAs found to be over-expressed in plasma of CRC patients in our study have also been investigated with respect to the other diseases in previous research [9], [10], [33], [35]–[48]. (Table 3) For example, plasma miR-21 was found to be over-expressed in a wide variety of malignancies including hepatocellular carcinoma, prostate cancer, and gastric cancer [35], [36], [42], [46]–[49]. These commonalities in miR-21 expression pattern in different cancers suggestes that miR-21 might function as an oncogene. Functional investigations showed that miR-21 targets tumor suppressor genes such as phosphatase and tensin homolog (PTEN) and inversely regulates its expression [50]. PTEN is ranked the second most commonly mutated tumor suppressor gene after p53. It can be inactivated by mutation with loss of heterozygosity, promoter methylation, miRNA interference and some other mechanisms in a number of cancers including brain, prostate, uterine cancer [51]. Differential expression of some miRNAs in multiple cancers support suggestions that they should typically be combined with additional miRNAs when used for detection of specific cancers.

**Table 3:** Circulating microRNAs involved in cancer and other diseases.

MiRNAs	Chromosomal location	Number of studies (references)	Expression in patients	Samples sizes	Expression of potential target gene
miR-18a	13q31.3	1 [42]	Up in pancreatic cancer	76	
miR-20a	13q31.3	2 [53]	Up in multiple myeloma	40	
miR-21	17q23.1	8 [35], [36], [42], [46]–[49]	Up in HCC	407	
			Up in ESCC (the miR-21/miR-375 ratio)	70	
			Up in NSCLC	93	
			Up in prostate cancer	71	
			Up in pancreatic cancer	60	PTEN, PDCD4, Maspin and TPM1 reduced
			Up in GC	96	
			Up in pancreatic cancer	85	
			Up in CHC	81	
miR-29a	7q32.3	2 [9], [40]	Up in CRC	159	
			Up in active pulmonary tuberculosis	127	
miR-92a	13q31.3	6 [9], [10], [37], [39], [43], [44], [54]	Up in CRC	159	
			Up in CRC	140	
			Down in HCC	20	
			Down in acute leukemia	77	
			Down in CRC	102	
			Down in CAD	53	
miR-106b	7q22.1	1 [42]	Up in GC	96	
miR-133a	18q11.2	2 [39], [45]	Up in CAD	53	
			Up in AMI	63	
miR-143	5q32	1 [38]	Up in enterovirus infection	40	
miR-145	5q32	1 [39]	Down in CAD	53	

Abbreviations: CRC, colorectal cancer; HCC, hepatocellular carcinoma; ESCC, oesophageal squamous cell carcinoma; NSCLC, non-small cell lung cancer; GC, gastric cancer; CAD, coronary artery disease; CHC, chronic hepatitis C virus infection; AMI, acute myocardial infarction; PTEN, phosphatase and tensin homolog; PDCD4, programmed cell death 4; TPM1, tropomyosin.

There are some limitations that need to be taken into account when interpreting the results of this study. First, the sample size is still small, especially in the marker selection phase. Second, a lot of miRNAs’ abundances in plasma are too low to be accurately quantified by qRT-PCR, therefore, some potential relevant markers could not be considered. Third, it remains to be determined to what extent modified expression levels of miRNAs found among CRC patients in this study are CRC specific.

### Conclusions

Plasma miRNAs appear to be potentially useful biomarkers for early detection and diagnosis of CRC. While research on plasma based miRNA profiling is still at very early stage compared to research on tissue based miRNA profiling, it might have the potential to contribute to development of new approaches of non-invasive, blood based CRC screening. Nevertheless, the diagnostic performance of the identified panel of miRNAs might not yet be sufficient to compete with performance of some other non-invasive tests, in particular immunochemical faecal occult blood tests [52]. Further research on a multi-marker blood based test, potentially including the panel of miRNAs identified in this study might be a promising approach to enhance the repertoire for non-invasive cancer screening.

## Supporting Information

Figure S1 **Receiver operating characteristic curves using 12 selected microRNAs (miR-18a, -20a, -21, -29a, -92a, -106b, -133a, -143, -145, -181b, -342-3p and miR-532-3p) for discrimination of 80 colorectal cancer patients and 144 neoplasm-free controls.** Abbreviations: FPR, false positive rate. TPR, true positive rate. AUC, area under receiver operating characteristic curve.
(DOC)

Table S1 **667 human microRNAs tested using TaqMan MicroRNA Array.**
(DOC)

Table S2 **RT-primer pools used for multiplex Real-Time quantitative PCR.**
(DOC)

Table S3 **Spearman correlation coefficients of microRNAs expression levels among 324 participants of the validation (p<0.001 if not annotated).**
(DOC)
