# Supplementary material for: Identification and Evaluation of Plasma MicroRNAs for Early Detection of Colorectal Cancer
Source: PLoS One. 2013 May 14;8(5):e62880. doi: 10.1371/journal.pone.0062880 (PMC3653912; doi:10.1371/journal.pone.0062880)
Supplement: Figure S1 — Receiver operating characteristic curves using 12 selected microRNAs (miR-18a, -20a, -21, -29a, -92a, -106b, -133a, -143, -145, -181b, -342-3p and miR-532-3p) for discrimination of 80 colorectal cancer patients and 144 neoplasm-free controls. Abbreviations: FPR, false positive rate. TPR, true positive rate. AUC, area under receiver operating characteristic curve. (DOC) [file pone.0062880.s001.doc]

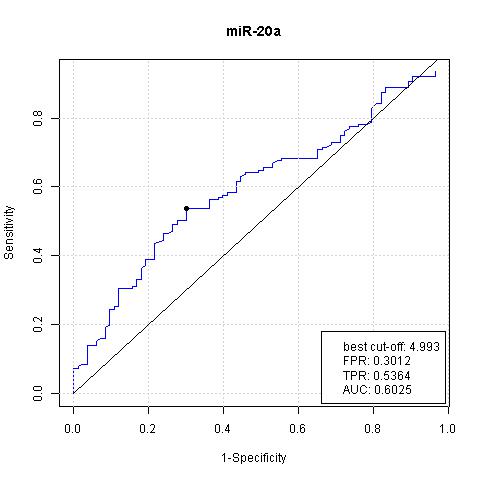

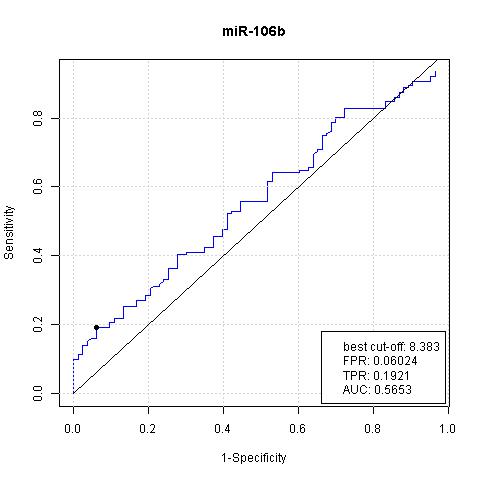

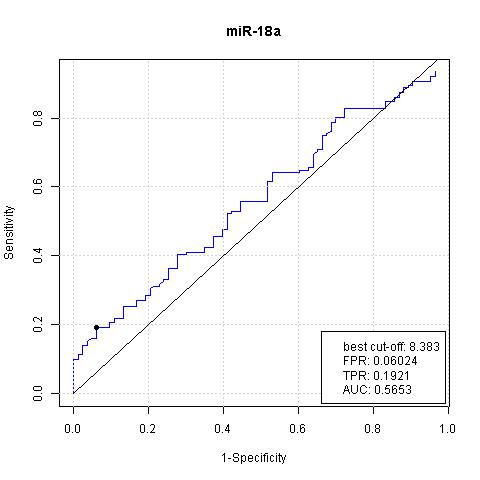

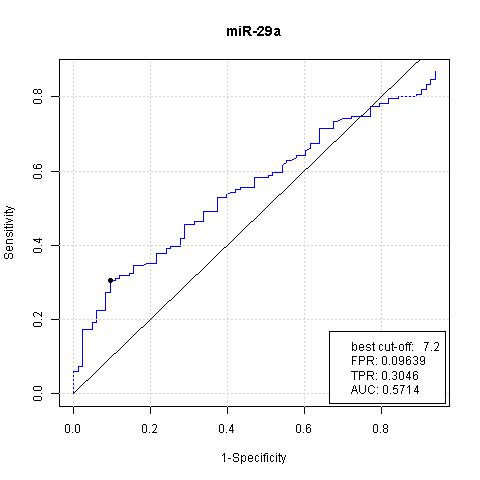

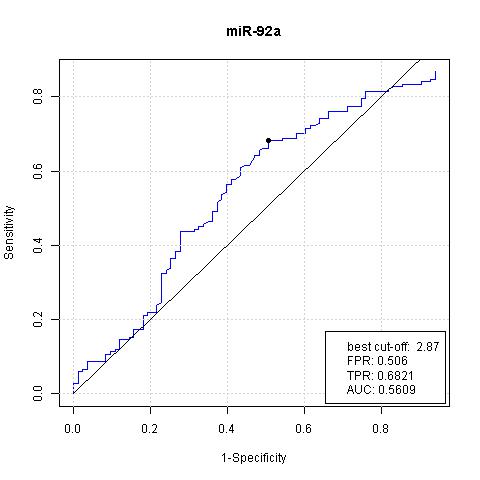

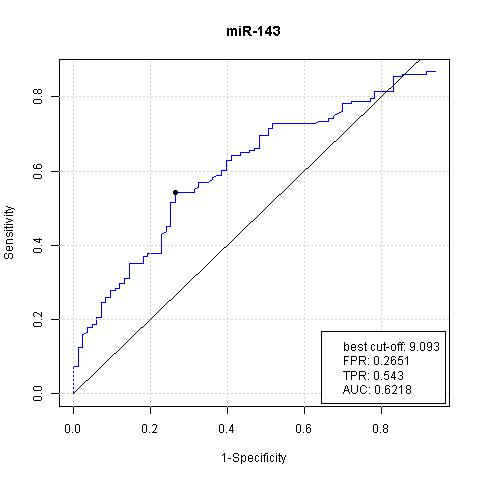

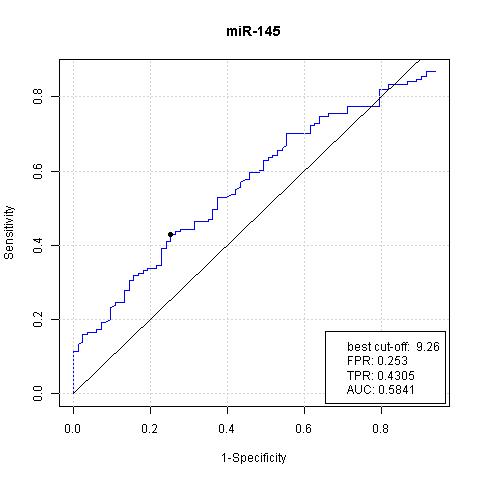

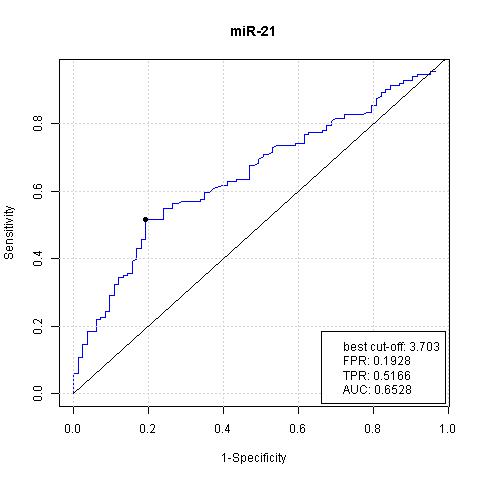

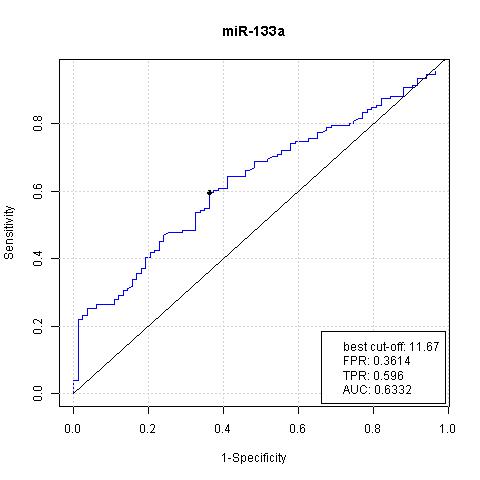

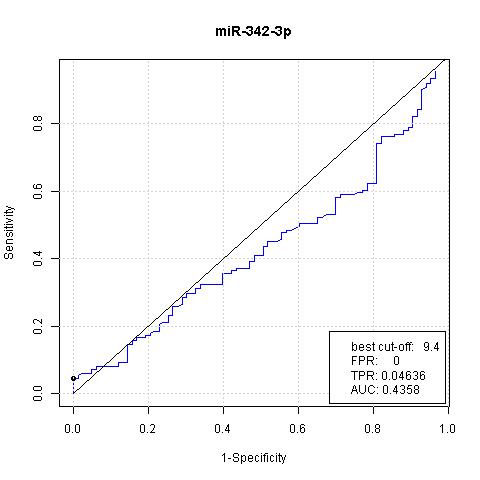

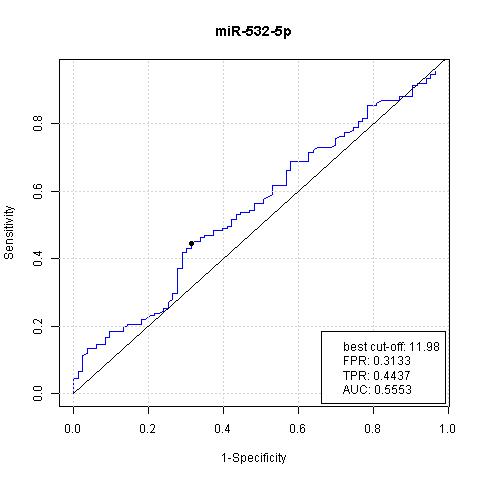

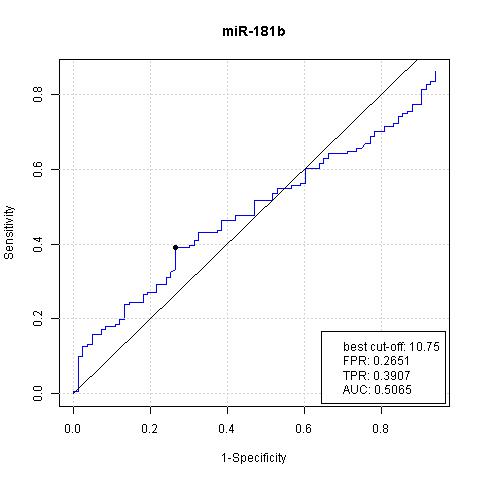


Figure S1. Receiver operating characteristic curves using 12 selected microRNAs (miR-18a, -20a, -21, -29a, -92a, -106b, -133a, -143, -145, -181b, -342-3p and miR-532-3p) for discrimination of 80 colorectal cancer patients and 144 neoplasm-free controls. Abbreviations: FPR, false positive rate. TPR, true positive rate. AUC, area under receiver operating characteristic curve.
