# Supplementary material for: Identification and Evaluation of Plasma MicroRNAs for Early Detection of Colorectal Cancer
Source: PLoS One. 2013 May 14;8(5):e62880. doi: 10.1371/journal.pone.0062880 (PMC3653912; doi:10.1371/journal.pone.0062880)
Supplement: Table S1 — 667 human microRNAs tested using TaqMan MicroRNA Array. (DOC) [file pone.0062880.s002.doc]

**Table S1**. 667 human microRNAs tested using TaqMan MicroRNA Array

| **miRNAs in card A** |  | **miRNAs in card B** |
| --- | --- | --- |
| hsa-miR-323-3p-4395338 |  | hsa-miR-135a*-4395343 |
| hsa-miR-597-4380960 |  | hsa-miR-138-1*-4395273 |
| hsa-miR-223-4395406 |  | hsa-miR-188-5p-4395431 |
| hsa-miR-222-4395387 |  | hsa-miR-877-4395402 |
| hsa-miR-548a-3p-4380948 |  | hsa-miR-610-4380980 |
| hsa-miR-518d-3p-4373248 |  | hsa-miR-760-4395439 |
| MammU6-4395470 |  | hsa-miR-632-4380977 |
| hsa-miR-636-4395199 |  | hsa-miR-30a-4373061 |
| MammU6-4395470 |  | hsa-miR-509-3p-4395347 |
| hsa-miR-212-4373087 |  | hsa-miR-645-4381000 |
| hsa-miR-133a-4395357 |  | hsa-miR-378-4395354 |
| hsa-miR-520f-4373256 |  | hsa-miR-516a-3p-4373183 |
| hsa-miR-30c-4373060 |  | hsa-miR-30e*-4373057 |
| MammU6-4395470 |  | hsa-miR-19b-1*-4395536 |
| hsa-miR-106b-4373155 |  | hsa-miR-645-4381000 |
| hsa-miR-582-3p-4395510 |  | hsa-miR-99b*-4395307 |
| hsa-miR-489-4395469 |  | hsa-miR-520c-3p-4395511 |
| hsa-miR-147-4373131 |  | hsa-miR-639-4380987 |
| hsa-let-7a-4373169 |  | hsa-miR-519b-3p-4395495 |
| hsa-let-7c-4373167 |  | MammU6-4395470 |
| hsa-let-7d-4395394 |  | hsa-miR-616*-4380992 |
| hsa-let-7e-4395517 |  | hsa-miR-661-4381009 |
| hsa-let-7f-4373164 |  | hsa-miR-378*-4373024 |
| hsa-let-7g-4395393 |  | MammU6-4395470 |
| hsa-miR-1-4395333 |  | hsa-miR-151-3p-4395365 |
| hsa-miR-9-4373285 |  | hsa-miR-656-4380920 |
| hsa-miR-10a-4373153 |  | hsa-miR-638-4380986 |
| hsa-miR-10b-4395329 |  | hsa-miR-623-4386740 |
| hsa-miR-15a-4373123 |  | hsa-miR-30a*-4373062 |
| hsa-miR-15b-4373122 |  | hsa-miR-34b*-4373037 |
| hsa-miR-16-4373121 |  | hsa-miR-126*-4373269 |
| hsa-miR-17-4395419 |  | hsa-miR-154*-4378065 |
| hsa-miR-18a-4395533 |  | hsa-miR-182*-4378066 |
| hsa-miR-18b-4395328 |  | hsa-miR-181a*-4373086 |
| hsa-miR-19a-4373099 |  | hsa-miR-302c*-4373277 |
| hsa-miR-19b-4373098 |  | hsa-miR-373*-4373279 |
| hsa-miR-20a-4373286 |  | MammU6-4395470 |
| hsa-miR-20b-4373263 |  | MammU6-4395470 |
| hsa-miR-21-4373090 |  | hsa-miR-380*-4373021 |
| hsa-miR-22-4373079 |  | hsa-miR-200a*-4373273 |
| hsa-miR-23a-4373074 |  | hsa-miR-432*-4378076 |
| hsa-miR-23b-4373073 |  | hsa-miR-493*-4373218 |
| hsa-miR-24-4373072 |  | hsa-miR-500*-4373225 |
| hsa-miR-25-4373071 |  | hsa-miR-488*-4373213 |
| hsa-miR-26a-4395166 |  | hsa-miR-517*-4378078 |
| hsa-miR-26b-4395167 |  | hsa-miR-518c*-4378082 |
| hsa-miR-27a-4373287 |  | hsa-miR-519e*-4378084 |
| hsa-miR-27b-4373068 |  | hsa-let-7d*-4378108 |
| hsa-miR-28-3p-4395557 |  | hsa-miR-363*-4380917 |
| hsa-miR-28-5p-4373067 |  | hsa-miR-7-1*-4381118 |
| MammU6-4395470 |  | hsa-miR-589*-4380953 |
| hsa-miR-29a-4395223 |  | hsa-miR-550*-4380954 |
| hsa-miR-29b-4373288 |  | hsa-miR-593*-4380957 |
| hsa-miR-29c-4395171 |  | hsa-miR-624*-4380964 |
| hsa-miR-30b-4373290 |  | hsa-miR-629*-4380969 |
| hsa-miR-31-4395390 |  | hsa-miR-29c*-4381131 |
| hsa-miR-32-4395220 |  | hsa-miR-454*-4395185 |
| hsa-miR-33b-4395196 |  | hsa-miR-505*-4395198 |
| hsa-miR-34a-4395168 |  | hsa-miR-221*-4395207 |
| hsa-miR-34c-5p-4373036 |  | hsa-miR-222*-4395208 |
| hsa-miR-92a-4395169 |  | hsa-miR-223*-4395209 |
| hsa-miR-93-4373302 |  | hsa-miR-136*-4395211 |
| hsa-miR-95-4373011 |  | hsa-miR-185*-4395215 |
| hsa-miR-96-4373372 |  | hsa-miR-186*-4395216 |
| hsa-miR-98-4373009 |  | hsa-miR-195*-4395218 |
| hsa-miR-99a-4373008 |  | hsa-miR-30c-1*-4395219 |
| hsa-miR-99b-4373007 |  | hsa-miR-30c-2*-4395221 |
| hsa-miR-100-4373160 |  | hsa-miR-32*-4395222 |
| hsa-miR-101-4395364 |  | hsa-miR-130b*-4395225 |
| hsa-miR-103-4373158 |  | hsa-miR-26a-2*-4395226 |
| hsa-miR-105-4395278 |  | hsa-let-7g*-4395229 |
| hsa-miR-106a-4395280 |  | hsa-miR-302b*-4395230 |
| RNU44-4373384 |  | hsa-miR-302d*-4395231 |
| hsa-miR-107-4373154 |  | hsa-miR-367*-4395232 |
| hsa-miR-122-4395356 |  | hsa-miR-374a*-4395236 |
| hsa-miR-124-4373295 |  | hsa-miR-23b*-4395237 |
| hsa-miR-125a-3p-4395310 |  | hsa-miR-376a*-4395238 |
| hsa-miR-125a-5p-4395309 |  | hsa-miR-377*-4395239 |
| hsa-miR-125b-4373148 |  | hsa-miR-30b*-4395240 |
| hsa-miR-126-4395339 |  | hsa-miR-122*-4395241 |
| hsa-miR-127-3p-4373147 |  | hsa-miR-130a*-4395242 |
| hsa-miR-127-5p-4395340 |  | RNU48-4373383 |
| hsa-miR-128-4395327 |  | RNU48-4373383 |
| hsa-miR-129-3p-4373297 |  | hsa-miR-132*-4395243 |
| hsa-miR-129-5p-4373171 |  | hsa-miR-379*-4395244 |
| hsa-miR-130a-4373145 |  | hsa-miR-148a*-4395245 |
| hsa-miR-130b-4373144 |  | hsa-miR-33a*-4395247 |
| hsa-miR-132-4373143 |  | hsa-miR-92a-1*-4395248 |
| hsa-miR-133b-4395358 |  | hsa-miR-92a-2*-4395249 |
| hsa-miR-134-4373299 |  | hsa-miR-93*-4395250 |
| hsa-miR-135a-4373140 |  | hsa-miR-96*-4395251 |
| hsa-miR-135b-4395372 |  | hsa-miR-99a*-4395252 |
| hsa-miR-136-4373173 |  | hsa-miR-100*-4395253 |
| hsa-miR-137-4373301 |  | hsa-miR-101*-4395254 |
| hsa-miR-138-4395395 |  | hsa-miR-138-2*-4395255 |
| hsa-miR-139-3p-4395424 |  | hsa-miR-141*-4395256 |
| hsa-miR-139-5p-4395400 |  | hsa-miR-143*-4395257 |
| hsa-miR-140-3p-4395345 |  | hsa-miR-144*-4395259 |
| hsa-miR-140-5p-4373374 |  | hsa-miR-145*-4395260 |
| hsa-miR-141-4373137 |  | hsa-miR-125b-2*-4395269 |
| hsa-miR-142-3p-4373136 |  | hsa-miR-135b*-4395270 |
| hsa-miR-142-5p-4395359 |  | hsa-miR-148b*-4395271 |
| hsa-miR-143-4395360 |  | hsa-miR-146a*-4395274 |
| hsa-miR-145-4395389 |  | hsa-miR-149*-4395275 |
| hsa-miR-146a-4373132 |  | RNU48-4373383 |
| hsa-miR-146b-3p-4395472 |  | RNU48-4373383 |
| hsa-miR-146b-5p-4373178 |  | hsa-miR-29b-1*-4395276 |
| hsa-miR-147b-4395373 |  | hsa-miR-29b-2*-4395277 |
| hsa-miR-148a-4373130 |  | hsa-miR-105*-4395279 |
| hsa-miR-148b-4373129 |  | hsa-miR-106a*-4395281 |
| hsa-miR-149-4395366 |  | hsa-miR-16-2*-4395282 |
| hsa-miR-150-4373127 |  | hsa-let-7i*-4395283 |
| hsa-miR-152-4395170 |  | hsa-miR-15b*-4395284 |
| hsa-miR-153-4373305 |  | hsa-miR-27b*-4395285 |
| hsa-miR-154-4373270 |  | hsa-miR-335*-4395296 |
| hsa-miR-181a-4373117 |  | hsa-miR-124*-4395308 |
| hsa-miR-181c-4373115 |  | hsa-miR-541*-4395311 |
| hsa-miR-182-4395445 |  | hsa-miR-888*-4395324 |
| RNU48-4373383 |  | hsa-miR-9*-4395342 |
| hsa-miR-183-4395380 |  | hsa-miR-411*-4395349 |
| hsa-miR-184-4373113 |  | hsa-miR-340*-4395370 |
| hsa-miR-185-4395382 |  | hsa-miR-545*-4395377 |
| hsa-miR-186-4395396 |  | hsa-miR-183*-4395381 |
| hsa-miR-187-4373307 |  | hsa-miR-192*-4395383 |
| hsa-miR-188-3p-4395217 |  | hsa-miR-200b*-4395385 |
| hsa-miR-190-4373110 |  | hsa-miR-200c*-4395397 |
| hsa-miR-191-4395410 |  | RNU44-4373384 |
| hsa-miR-192-4373108 |  | RNU44-4373384 |
| hsa-miR-193a-3p-4395361 |  | hsa-miR-155*-4395398 |
| hsa-miR-193a-5p-4395392 |  | hsa-miR-10a*-4395399 |
| hsa-miR-193b-4395478 |  | hsa-miR-214*-4395404 |
| hsa-miR-194-4373106 |  | hsa-miR-218-2*-4395405 |
| hsa-miR-195-4373105 |  | hsa-miR-22*-4395412 |
| hsa-miR-196b-4395326 |  | hsa-miR-425*-4395413 |
| hsa-miR-197-4373102 |  | hsa-miR-30d*-4395416 |
| hsa-miR-198-4395384 |  | hsa-let-7a*-4395418 |
| hsa-miR-199a-5p-4373272 |  | hsa-miR-424*-4395420 |
| hsa-miR-199a-3p-4395415 |  | hsa-miR-18b*-4395421 |
| hsa-miR-199b-5p-4373100 |  | hsa-miR-20b*-4395422 |
| hsa-miR-200a-4378069 |  | hsa-miR-431*-4395423 |
| hsa-miR-200b-4395362 |  | hsa-miR-7-2*-4395425 |
| hsa-miR-200c-4395411 |  | hsa-miR-10b*-4395426 |
| hsa-miR-202-4395474 |  | hsa-miR-34a*-4395427 |
| hsa-miR-203-4373095 |  | hsa-miR-181a-2*-4395428 |
| hsa-miR-204-4373094 |  | hsa-miR-744*-4395436 |
| hsa-miR-205-4373093 |  | hsa-miR-452*-4395441 |
| hsa-miR-208b-4395401 |  | hsa-miR-181c*-4395444 |
| hsa-miR-210-4373089 |  | hsa-miR-708*-4395453 |
| hsa-miR-214-4395417 |  | hsa-miR-92b*-4395454 |
| hsa-miR-215-4373084 |  | hsa-miR-551b*-4395457 |
| hsa-miR-216a-4395331 |  | RNU44-4373384 |
| hsa-miR-216b-4395437 |  | RNU44-4373384 |
| hsa-miR-217-4395448 |  | hsa-miR-202*-4395473 |
| hsa-miR-218-4373081 |  | hsa-miR-193b*-4395477 |
| hsa-miR-219-5p-4373080 |  | hsa-miR-497*-4395479 |
| hsa-miR-221-4373077 |  | hsa-miR-518e*-4395482 |
| hsa-miR-224-4395210 |  | hsa-miR-125b-1*-4395489 |
| hsa-miR-296-3p-4395212 |  | hsa-miR-194*-4395490 |
| hsa-miR-296-5p-4373066 |  | hsa-miR-106b*-4395491 |
| hsa-miR-299-3p-4373189 |  | hsa-miR-302a*-4395492 |
| hsa-miR-299-5p-4373188 |  | hsa-miR-526b*-4395494 |
| hsa-miR-301a-4373064 |  | hsa-miR-518f*-4395498 |
| hsa-miR-301b-4395503 |  | hsa-miR-374b*-4395502 |
| hsa-miR-302a-4378070 |  | hsa-let-7b*-4395515 |
| ath-miR159a-4373390 |  | hsa-let-7e*-4395518 |
| hsa-miR-302b-4378071 |  | hsa-let-7f-1*-4395528 |
| hsa-miR-302c-4378072 |  | hsa-let-7f-2*-4395529 |
| hsa-miR-320-4395388 |  | hsa-miR-15a*-4395530 |
| hsa-miR-324-3p-4395272 |  | hsa-miR-16-1*-4395531 |
| hsa-miR-324-5p-4373052 |  | hsa-miR-17*-4395532 |
| hsa-miR-326-4373050 |  | hsa-miR-18a*-4395534 |
| hsa-miR-328-4373049 |  | hsa-miR-19a*-4395535 |
| hsa-miR-329-4373191 |  | hsa-miR-19b-2*-4395537 |
| hsa-miR-330-3p-4373047 |  | RNU24-4373379 |
| hsa-miR-330-5p-4395341 |  | RNU24-4373379 |
| hsa-miR-331-3p-4373046 |  | hsa-miR-625*-4395543 |
| hsa-miR-331-5p-4395344 |  | hsa-miR-20a*-4395548 |
| hsa-miR-335-4373045 |  | hsa-miR-21*-4395549 |
| hsa-miR-337-5p-4395267 |  | hsa-miR-23a*-4395550 |
| hsa-miR-338-3p-4395363 |  | hsa-miR-24-1*-4395551 |
| hsa-miR-339-3p-4395295 |  | hsa-miR-25*-4395553 |
| hsa-miR-339-5p-4395368 |  | hsa-miR-26a-1*-4395554 |
| hsa-miR-340-4395369 |  | hsa-miR-26b*-4395555 |
| has-miR-155-4395459 |  | hsa-miR-27a*-4395556 |
| hsa-let-7b-4395446 |  | hsa-miR-29a*-4395558 |
| hsa-miR-342-3p-4395371 |  | hsa-miR-944-4395300 |
| hsa-miR-342-5p-4395258 |  | hsa-miR-943-4395299 |
| hsa-miR-345-4395297 |  | hsa-miR-942-4395298 |
| hsa-miR-361-5p-4373035 |  | hsa-miR-941-4395294 |
| hsa-miR-362-3p-4395228 |  | hsa-miR-939-4395293 |
| hsa-miR-362-5p-4378092 |  | hsa-miR-938-4395292 |
| hsa-miR-363-4378090 |  | hsa-miR-937-4395291 |
| hsa-miR-365-4373194 |  | hsa-miR-936-4395290 |
| hsa-miR-367-4373034 |  | hsa-miR-892b-4395325 |
| hsa-miR-369-3p-4373032 |  | hsa-miR-875-5p-4395314 |
| hsa-miR-369-5p-4373195 |  | hsa-miR-770-5p-4395189 |
| hsa-miR-370-4395386 |  | RNU24-4373379 |
| hsa-miR-371-3p-4395235 |  | RNU24-4373379 |
| hsa-miR-372-4373029 |  | hsa-miR-659-4380924 |
| hsa-miR-373-4378073 |  | hsa-miR-643-4380997 |
| hsa-miR-374a-4373028 |  | hsa-miR-630-4380970 |
| hsa-miR-374b-4381045 |  | hsa-miR-619-4380998 |
| hsa-miR-375-4373027 |  | hsa-miR-617-4380994 |
| hsa-miR-376a-4373026 |  | hsa-miR-612-4380983 |
| hsa-miR-376b-4373196 |  | hsa-miR-603-4380972 |
| hsa-miR-377-4373025 |  | hsa-miR-601-4380965 |
| hsa-miR-379-4373349 |  | hsa-miR-595-4395178 |
| hsa-miR-380-4373022 |  | hsa-miR-593-4395522 |
| hsa-miR-381-4373020 |  | hsa-miR-592-4380956 |
| hsa-miR-382-4373019 |  | hsa-miR-587-4380950 |
| hsa-miR-383-4373018 |  | hsa-miR-586-4380949 |
| hsa-miR-409-5p-4395442 |  | hsa-miR-585-4381027 |
| hsa-miR-410-4378093 |  | hsa-miR-583-4381025 |
| hsa-miR-411-4381013 |  | hsa-miR-575-4381020 |
| hsa-miR-422a-4395408 |  | hsa-miR-573-4381018 |
| hsa-miR-423-5p-4395451 |  | RNU43-4373375 |
| hsa-miR-424-4373201 |  | RNU43-4373375 |
| hsa-miR-425-4380926 |  | RNU6B-4373381 |
| hsa-miR-429-4373203 |  | RNU6B-4373381 |
| hsa-miR-431-4395173 |  | hsa-miR-569-4380946 |
| hsa-miR-433-4373205 |  | hsa-miR-567-4380944 |
| hsa-miR-449a-4373207 |  | hsa-miR-565-4380942 |
| hsa-miR-449b-4381011 |  | hsa-miR-564-4380941 |
| hsa-miR-450a-4395414 |  | hsa-miR-563-4380940 |
| hsa-miR-450b-3p-4395319 |  | hsa-miR-558-4380936 |
| hsa-miR-450b-5p-4395318 |  | hsa-miR-557-4380935 |
| hsa-miR-451-4373360 |  | hsa-miR-555-4380933 |
| hsa-miR-452-4395440 |  | hsa-miR-554-4380932 |
| hsa-miR-453-4395429 |  | hsa-miR-553-4380931 |
| hsa-miR-454-4395434 |  | hsa-miR-552-4380930 |
| hsa-miR-455-3p-4395355 |  | hsa-miR-551a-4380929 |
| hsa-miR-455-5p-4378098 |  | hsa-miR-550-4395521 |
| hsa-miR-483-5p-4395449 |  | hsa-miR-549-4380921 |
| hsa-miR-484-4381032 |  | hsa-miR-543-4395487 |
| hsa-miR-485-3p-4378095 |  | hsa-miR-520h-4373258 |
| hsa-miR-485-5p-4373212 |  | hsa-miR-513-3p-4395202 |
| hsa-miR-486-3p-4395204 |  | RNU43-4373375 |
| hsa-miR-486-5p-4378096 |  | RNU43-4373375 |
| hsa-miR-487a-4378097 |  | RNU6B-4373381 |
| hsa-miR-487b-4378102 |  | RNU6B-4373381 |
| hsa-miR-488-4395468 |  | ath-miR159a-4373390 |
| hsa-miR-490-3p-4373215 |  | hsa-miR-498-4373223 |
| hsa-miR-491-3p-4395471 |  | hsa-miR-337-3p-4395268 |
| hsa-miR-491-5p-4381053 |  | hsa-miR-30e-4395334 |
| hsa-miR-493-4395475 |  | hsa-miR-206-4373092 |
| hsa-miR-494-4395476 |  | hsa-miR-190b-4395374 |
| hsa-miR-495-4381078 |  | hsa-miR-935-4395289 |
| hsa-miR-496-4386771 |  | hsa-miR-934-4395288 |
| hsa-miR-499-3p-4395538 |  | hsa-miR-933-4395287 |
| hsa-miR-499-5p-4381047 |  | hsa-miR-924-4395265 |
| hsa-miR-500-4395539 |  | hsa-miR-923-4395264 |
| hsa-miR-501-3p-4395546 |  | hsa-miR-922-4395263 |
| hsa-miR-501-5p-4373226 |  | hsa-miR-921-4395262 |
| hsa-miR-502-3p-4395194 |  | hsa-miR-920-4395261 |
| hsa-miR-502-5p-4373227 |  | hsa-miR-801-4395183 |
| hsa-miR-503-4373228 |  | hsa-miR-769-5p-4395186 |
| hsa-miR-504-4395195 |  | hsa-miR-769-3p-4395190 |
| hsa-miR-505-4395200 |  | hsa-miR-768-3p-4395188 |
| hsa-miR-507-4373232 |  | hsa-miR-767-5p-4395182 |
| hsa-miR-508-3p-4373233 |  | hsa-miR-767-3p-4395184 |
| hsa-miR-508-5p-4395203 |  | hsa-miR-766-4395177 |
| hsa-miR-509-5p-4395346 |  | hsa-miR-7-4378130 |
| hsa-miR-510-4395352 |  | hsa-miR-675-4395192 |
| hsa-miR-512-3p-4381034 |  | hsa-miR-668-4395181 |
| hsa-miR-512-5p-4373238 |  | hsa-miR-662-4381010 |
| hsa-miR-513-5p-4395201 |  | hsa-miR-658-4380923 |
| hsa-miR-515-3p-4395480 |  | hsa-miR-657-4380922 |
| hsa-miR-515-5p-4373242 |  | hsa-miR-656-4380920 |
| hsa-miR-516a-5p-4395527 |  | hsa-miR-650-4381006 |
| hsa-miR-516b-4395172 |  | hsa-miR-649-4381005 |
| hsa-miR-517a-4395513 |  | hsa-miR-935-4395289 |
| hsa-miR-517c-4373264 |  | hsa-miR-934-4395288 |
| hsa-miR-518a-3p-4395508 |  | hsa-miR-933-4395287 |
| hsa-miR-518a-5p-4395507 |  | hsa-miR-924-4395265 |
| hsa-miR-518b-4373246 |  | hsa-miR-923-4395264 |
| hsa-miR-518c-4395512 |  | hsa-miR-922-4395263 |
| hsa-miR-518d-5p-4395500 |  | hsa-miR-921-4395262 |
| hsa-miR-518e-4395506 |  | hsa-miR-920-4395261 |
| hsa-miR-518f-4395499 |  | hsa-miR-801-4395183 |
| hsa-miR-519a-4395526 |  | hsa-miR-769-5p-4395186 |
| hsa-miR-519d-4395514 |  | hsa-miR-769-3p-4395190 |
| hsa-miR-519e-4395481 |  | hsa-miR-768-3p-4395188 |
| hsa-miR-520a-3p-4373268 |  | hsa-miR-767-5p-4395182 |
| hsa-miR-520a-5p-4378085 |  | hsa-miR-767-3p-4395184 |
| hsa-miR-520d-5p-4395504 |  | hsa-miR-766-4395177 |
| hsa-miR-520g-4373257 |  | hsa-miR-7-4378130 |
| hsa-miR-521-4373259 |  | hsa-miR-675-4395192 |
| hsa-miR-522-4395524 |  | hsa-miR-668-4395181 |
| hsa-miR-523-4395497 |  | hsa-miR-662-4381010 |
| hsa-miR-524-5p-4395174 |  | hsa-miR-658-4380923 |
| hsa-miR-525-3p-4395496 |  | hsa-miR-657-4380922 |
| hsa-miR-525-5p-4378088 |  | hsa-miR-650-4381006 |
| hsa-miR-526b-4395493 |  | hsa-miR-649-4381005 |
| hsa-miR-532-3p-4395466 |  | hsa-miR-648-4381004 |
| hsa-miR-532-5p-4380928 |  | hsa-miR-647-4381003 |
| hsa-miR-539-4378103 |  | hsa-miR-646-4381002 |
| hsa-miR-541-4395312 |  | hsa-miR-644-4380999 |
| hsa-miR-542-3p-4378101 |  | hsa-miR-641-4380988 |
| hsa-miR-542-5p-4395351 |  | hsa-miR-640-4386743 |
| hsa-miR-544-4395376 |  | hsa-miR-639-4380987 |
| hsa-miR-545-4395378 |  | hsa-miR-637-4380985 |
| hsa-miR-548a-5p-4395523 |  | hsa-miR-635-4380982 |
| hsa-miR-548b-3p-4380951 |  | hsa-miR-634-4380981 |
| hsa-miR-548b-5p-4395519 |  | hsa-miR-633-4380979 |
| hsa-miR-548c-3p-4380993 |  | hsa-miR-631-4380971 |
| hsa-miR-548c-5p-4395540 |  | hsa-miR-628-3p-4395545 |
| hsa-miR-548d-3p-4381008 |  | hsa-miR-626-4380966 |
| hsa-miR-548d-5p-4395348 |  | hsa-miR-623-4386740 |
| hsa-miR-551b-4380945 |  | hsa-miR-622-4380961 |
| hsa-miR-556-3p-4395456 |  | hsa-miR-621-4381001 |
| hsa-miR-556-5p-4395455 |  | hsa-miR-614-4380990 |
| hsa-miR-561-4380938 |  | hsa-miR-613-4380989 |
| hsa-miR-570-4395458 |  | hsa-miR-609-4380978 |
| hsa-miR-574-3p-4395460 |  | hsa-miR-608-4380976 |
| hsa-miR-576-3p-4395462 |  | hsa-miR-607-4380975 |
| hsa-miR-576-5p-4395461 |  | hsa-miR-606-4380974 |
| hsa-miR-579-4395509 |  | hsa-miR-605-4386742 |
| hsa-miR-582-5p-4395175 |  | hsa-miR-604-4380973 |
| hsa-miR-589-4395520 |  | hsa-miR-600-4380963 |
| hsa-miR-590-5p-4395176 |  | hsa-miR-599-4380962 |
| hsa-miR-598-4395179 |  | hsa-miR-596-4380959 |
| hsa-miR-615-3p-4386777 |  | hsa-miR-591-4380955 |
| hsa-miR-615-5p-4395464 |  | hsa-miR-588-4380952 |
| hsa-miR-616-4395525 |  | hsa-miR-584-4381026 |
| hsa-miR-618-4380996 |  | hsa-miR-581-4386744 |
| hsa-miR-624-4395541 |  | hsa-miR-580-4381024 |
| hsa-miR-625-4395542 |  | hsa-miR-578-4381022 |
| hsa-miR-627-4380967 |  | hsa-miR-572-4381017 |
| hsa-miR-628-5p-4395544 |  | hsa-miR-571-4381016 |
| hsa-miR-629-4395547 |  | hsa-miR-566-4380943 |
| hsa-miR-642-4380995 |  | hsa-miR-562-4380939 |
| hsa-miR-651-4381007 |  | hsa-miR-559-4380937 |
| hsa-miR-652-4395463 |  | hsa-miR-524-3p-4378087 |
| hsa-miR-653-4395403 |  | hsa-miR-519b-3p-4395495 |
| hsa-miR-654-3p-4395350 |  | hsa-miR-497-4373222 |
| hsa-miR-654-5p-4381014 |  | hsa-miR-432-4373280 |
| hsa-miR-655-4381015 |  | hsa-miR-409-3p-4395443 |
| hsa-miR-660-4380925 |  | hsa-miR-361-3p-4395227 |
| hsa-miR-671-3p-4395433 |  | hsa-miR-30d-4373059 |
| hsa-miR-672-4395438 |  | hsa-miR-302d-4373063 |
| hsa-miR-674-4395193 |  | hsa-miR-648-4381004 |
| hsa-miR-708-4395452 |  | hsa-miR-647-4381003 |
| hsa-miR-744-4395435 |  | hsa-miR-646-4381002 |
| hsa-miR-758-4395180 |  | hsa-miR-644-4380999 |
| hsa-miR-871-4395465 |  | hsa-miR-641-4380988 |
| hsa-miR-872-4395375 |  | hsa-miR-640-4386743 |
| hsa-miR-873-4395467 |  | hsa-miR-637-4380985 |
| hsa-miR-874-4395379 |  | hsa-miR-635-4380982 |
| hsa-miR-875-3p-4395315 |  | hsa-miR-634-4380981 |
| hsa-miR-876-3p-4395336 |  | hsa-miR-633-4380979 |
| hsa-miR-876-5p-4395316 |  | hsa-miR-631-4380971 |
| hsa-miR-885-3p-4395483 |  | hsa-miR-628-3p-4395545 |
| hsa-miR-885-5p-4395407 |  | hsa-miR-626-4380966 |
| hsa-miR-886-3p-4395305 |  | hsa-miR-622-4380961 |
| hsa-miR-886-5p-4395304 |  | hsa-miR-621-4381001 |
| hsa-miR-887-4395485 |  | hsa-miR-614-4380990 |
| hsa-miR-888-4395323 |  | hsa-miR-613-4380989 |
| hsa-miR-889-4395313 |  | hsa-miR-609-4380978 |
| hsa-miR-890-4395320 |  | hsa-miR-608-4380976 |
| hsa-miR-891a-4395302 |  | hsa-miR-607-4380975 |
| hsa-miR-891b-4395321 |  | hsa-miR-606-4380974 |
| hsa-miR-892a-4395306 |  | hsa-miR-605-4386742 |
| hsa-miR-208-4373091 |  | hsa-miR-604-4380973 |
| hsa-miR-211-4373088 |  | hsa-miR-600-4380963 |
| hsa-miR-219-1-3p-4395206 |  | hsa-miR-599-4380962 |
| hsa-miR-219-2-3p-4395501 |  | hsa-miR-596-4380959 |
| hsa-miR-220-4373078 |  | hsa-miR-591-4380955 |
| hsa-miR-220b-4395317 |  | hsa-miR-588-4380952 |
| hsa-miR-220c-4395322 |  | hsa-miR-584-4381026 |
| hsa-miR-298-4395301 |  | hsa-miR-581-4386744 |
| hsa-miR-325-4373051 |  | hsa-miR-580-4381024 |
| hsa-miR-346-4373038 |  | hsa-miR-578-4381022 |
| hsa-miR-376c-4395233 |  | hsa-miR-572-4381017 |
| hsa-miR-384-4373017 |  | hsa-miR-571-4381016 |
| hsa-miR-412-4373199 |  | hsa-miR-566-4380943 |
| hsa-miR-448-4373206 |  | hsa-miR-562-4380939 |
| hsa-miR-492-4373217 |  | hsa-miR-559-4380937 |
| hsa-miR-506-4373231 |  | hsa-miR-524-3p-4378087 |
| hsa-miR-509-3-5p-4395266 |  | hsa-miR-497-4373222 |
| hsa-miR-511-4373236 |  | hsa-miR-432-4373280 |
| hsa-miR-517b-4373244 |  | hsa-miR-409-3p-4395443 |
| hsa-miR-519c-3p-4373251 |  | hsa-miR-361-3p-4395227 |
| hsa-miR-520b-4373252 |  | hsa-miR-30d-4373059 |
| hsa-miR-520e-4373255 |  | hsa-miR-302d-4373063 |
