# Supplementary material for: Identification and Evaluation of Plasma MicroRNAs for Early Detection of Colorectal Cancer
Source: PLoS One. 2013 May 14;8(5):e62880. doi: 10.1371/journal.pone.0062880 (PMC3653912; doi:10.1371/journal.pone.0062880)
Supplement: Table S2 — RT-primer pools used for multiplex Real-Time quantitative PCR. (DOC) [file pone.0062880.s003.doc]

**Table S2**. RT-primer pools used for multiplex Real-Time quantitative PCR.

| **Multiplex sets** | **Set 1** | **Set 2** | **Set 3** |
| --- | --- | --- | --- |
| Endogenous control | miR-16/ RNU6B | miR-16/ RNU6B | miR-16/ RNU6B |
| Spiked-in control | cel-miR-39 |  |  |
| High expression | miR-20a | miR-92a | miR-21 |
| Moderate expression | miR-106b | miR-29a | miR-342-3p |
|  |  | miR-181b |  |
| Low expression | miR-18a | miR-143 | miR-133a |
|  |  | miR-145 | miR-532-3p |
