# Supplementary material for: Identification and Evaluation of Plasma MicroRNAs for Early Detection of Colorectal Cancer
Source: PLoS One. 2013 May 14;8(5):e62880. doi: 10.1371/journal.pone.0062880 (PMC3653912; doi:10.1371/journal.pone.0062880)
Supplement: Table S3 — Spearman correlation coefficients of microRNAs expression levels among 324 participants of the validation (p<0.001 if not annotated). (DOC) [file pone.0062880.s004.doc]

Table S3. Spearman correlation coefficients of microRNAs expression levels among 324 participants of the validation (p<0.001 if not annotated).

|  | miR-18a | miR-20a | miR-21 | miR-29a | miR-92a | miR-106b | miR-133a | miR-143 | miR-145 |
| --- | --- | --- | --- | --- | --- | --- | --- | --- | --- |
| miR-18a |  |  |  |  |  |  |  |  |  |
| miR-20a | 0.800 |  |  |  |  |  |  |  |  |
| miR-21 | 0.780 | 0.733 |  |  |  |  |  |  |  |
| miR-29a | 0.659 | 0.702 | 0.766 |  |  |  |  |  |  |
| miR-92a | 0.340 | 0.250 | 0.352 | 0.090  P=0.114 |  |  |  |  |  |
| miR-106b | 0.865 | 0.791 | 0.709 | 0.695 | 0.275 |  |  |  |  |
| miR-133a | 0.617 | 0.640 | 0.588 | 0.486 | 0.285 | 0.523 |  |  |  |
| miR-143 | 0.572 | 0.389 | 0.628 | 0.604 | 0.225 | 0.481 | 0.434 |  |  |
| miR-145 | 0.725 | 0.637 | 0.690 | 0.627 | 0.355 | 0.702 | 0.583 | 0.764 |  |
